# Coverage of necrosis after secondary cranioplasty using a latissimus dorsi muscle flap

**DOI:** 10.1093/jscr/rjaf1042

**Published:** 2026-01-08

**Authors:** Julian Ramin Andresen, Wolfgang Happak, Magnus Kuess, Harald Kurt Widhalm

**Affiliations:** Division of Trauma Surgery, Department of Orthopaedics and Trauma Surgery, Medical University of Vienna, Vienna, Austria; Department of Plastic and Reconstructive Surgery, Medical University of Vienna, Vienna, Austria; Department of Neurosurgery, Medical University of Vienna, Vienna, Austria; Division of Trauma Surgery, Department of Orthopaedics and Trauma Surgery, Medical University of Vienna, Vienna, Austria

**Keywords:** cranioplasty, head injury, musculus latissimus dorsi flap, scalp defect

## Abstract

Secondary cranioplasty aims to restore skull integrity after decompressive surgery but can be complicated by rare issues such as skin necrosis. We report a case of a 70-year-old female with multiple comorbidities who developed extensive scalp necrosis following cranioplasty. Surgical revision revealed significant dural exposure, requiring reconstruction with a free latissimus dorsi flap and a split-thickness skin grafting. Microvascular anastomosis was performed using the superficial temporal vessels. At 8-week follow-up, the site was fully healed without infection or recurrent necrosis. This case underscores the effectiveness of the latissimus dorsi free flap in managing large cranial defects, especially in patients with complex vascular conditions.

## Introduction

Secondary cranioplasty represents a complex and often challenging procedure for the reconstructive surgeon. It is typically performed to correct scalp and cranial defects following a prior craniectomy, which may have been necessary due to traumatic brain injury, hemorrhagic, or ischemic stroke, tumor resection, or congenital cranial deformities [1]. These procedures serve to relieve elevated intracranial pressure and offer neuroprotection, while also aiming to restore the anatomical integrity of the skull [2].

Although secondary cranioplasty is generally performed without major complications, cases involving vascular injury during the initial intervention - such as damage to the sinus sagittalis superior - can compromise scalp perfusion and result in rare but serious complications such as skin necrosis.

In cases of intraoperative dual compromise, the latissimus dorsi free flap represents a reliable reconstructive option. This flap has broad applications in reconstructive surgery, including in the head and neck, breast, and trauma settings [3, 4].

Recent advances include the use of patient-specific, 3D-printed implants composed of titanium or polymethylmethacrylate (PMMA), which improve anatomical fit and long-term outcomes [5–7]. These implants can be combined with vascularized tissue flaps to optimize both structural and esthetic results. Long-term studies have demonstrated high patient satisfaction and reduced complication rates with such hybrid approaches [8].

Moreover, intraoperative indocyanine green fluorescence angiography has emerged as a valuable tool for real-time assessment of flap perfusion, facilitating the selection of reliable perforators and verification of microvascular anastomotic patency [9–11].

## Case report

### Patient history and presentation

A 70-year-old woman presented to the emergency department following a head injury sustained from a fall. Despite the trauma, she was fully conscious and did not report any episodes of vomiting. Her medical history included hypertension, atrial fibrillation, and Parkinson’s disease. She had also been on long-term anticoagulation therapy with phenprocoumon.

### Initial assessment

**Figure 1:**
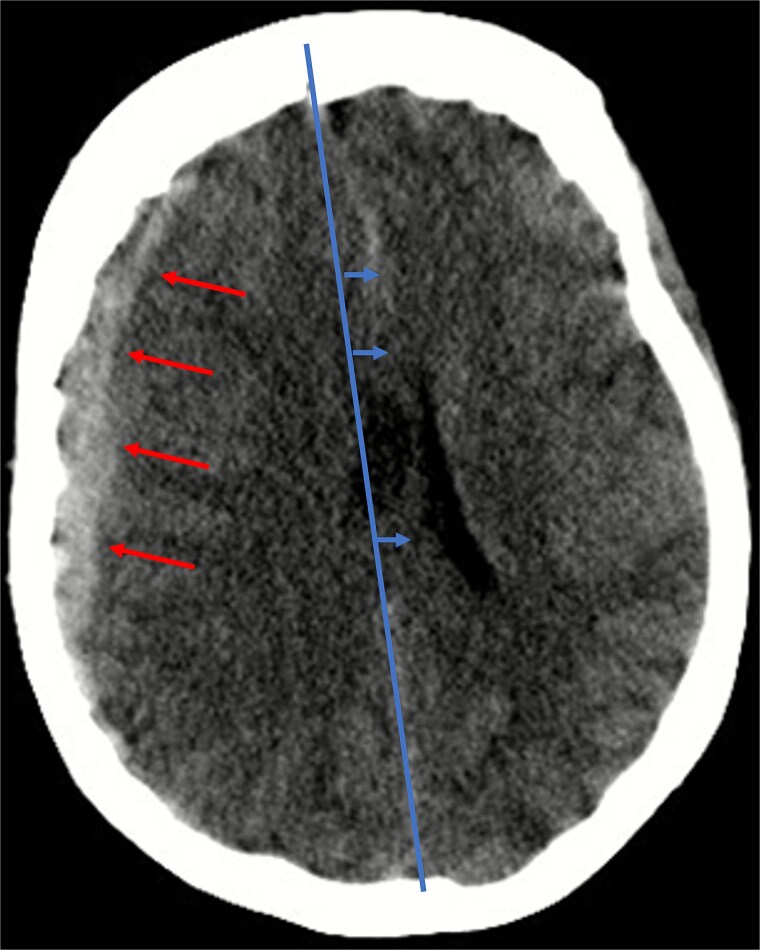
Illustration of a hyperdense, concave 13 mm wide mass as an expression of a subdural hematoma in the right hemispheric region (red arrows) and a consecutive midline shift of 8 mm due to edema (blue arrows).

Given her anticoagulated state, a cranial computed tomography (CT) scan was performed, in line with departmental protocols for patients with head trauma on anticoagulation. The scan revealed a 13 mm-wide subdural hematoma spanning the entire right hemisphere, accompanied by an 8 mm midline shift, and near-complete collapse of the right lateral ventricle (Fig. 1). Additionally, diffuse cerebral swelling was also present. There were no signs of skull fractures or ischemia.

### Surgical intervention

**Figure 2:**
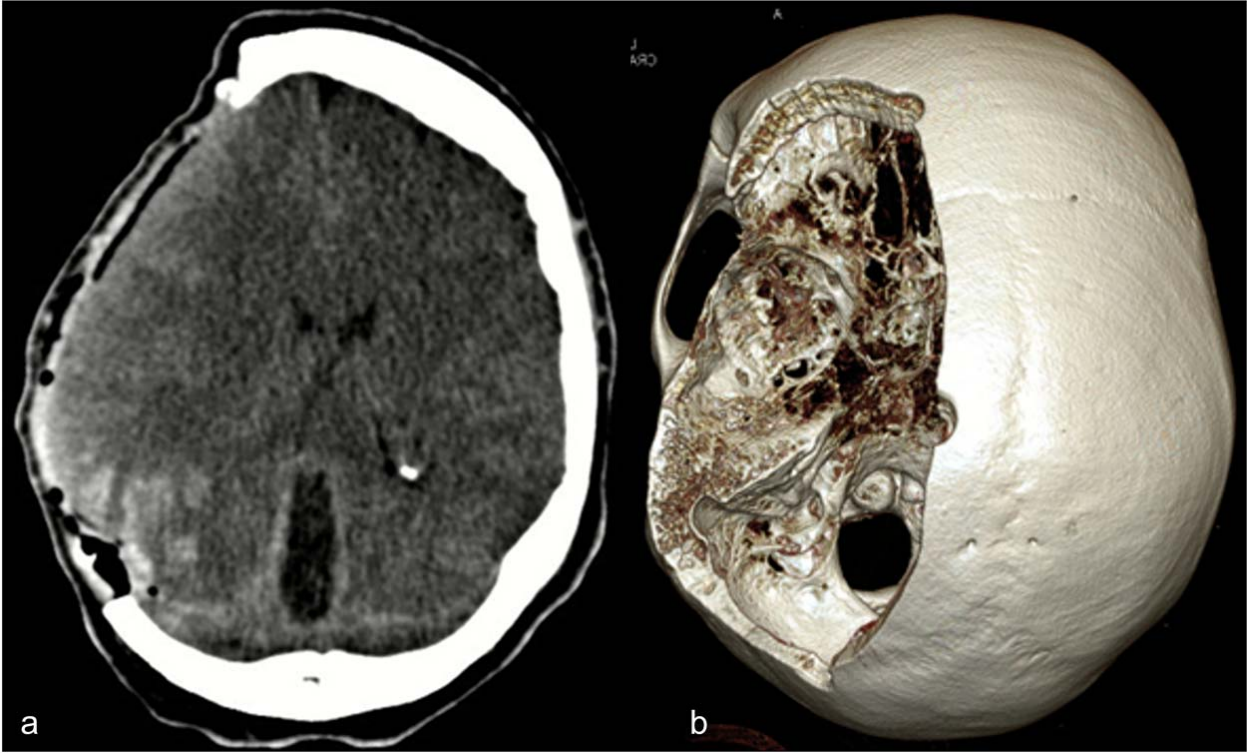
(a) Axial CT image after osteoclastic craniotomy and (b) in the 3D reconstruction representation of the large bone defect.

**Figure 3:**
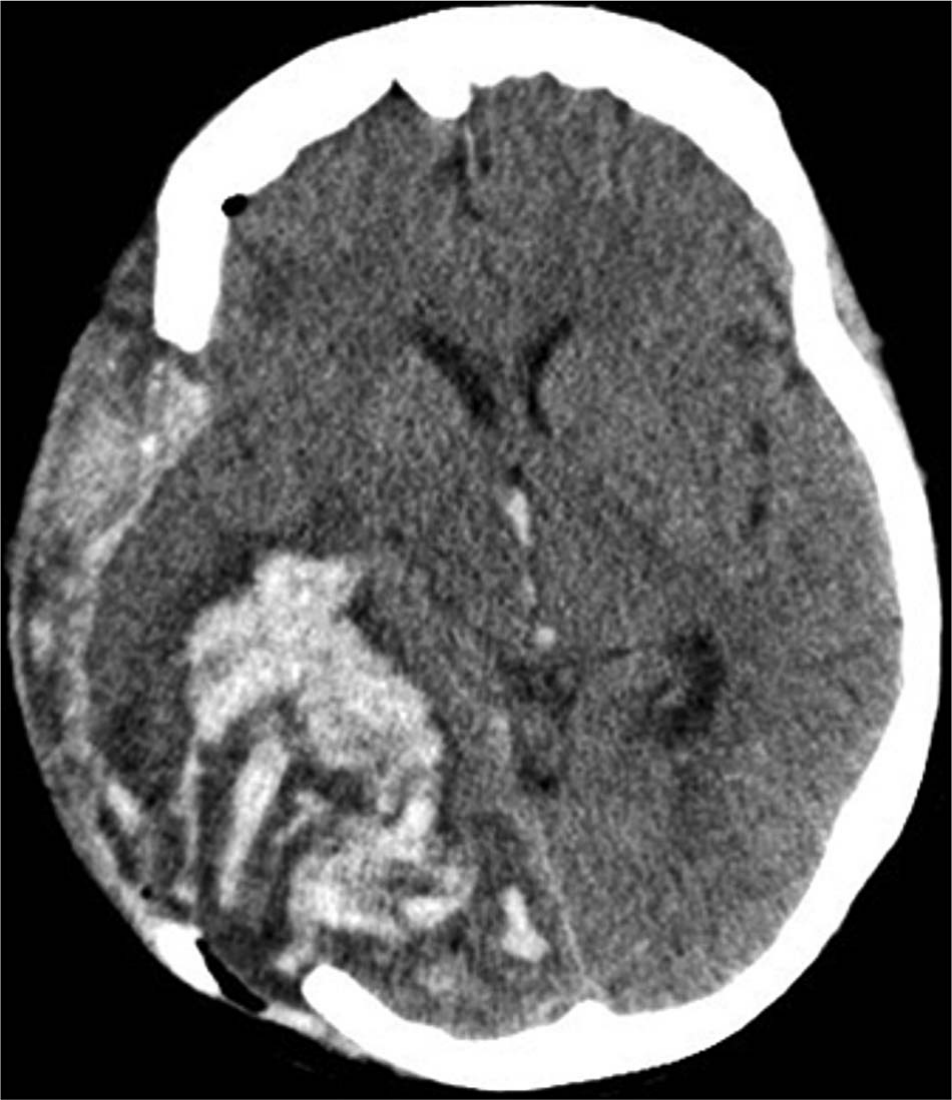
Postoperative axial CT scan. After osteoclastic craniotomy, a large bony defect with parenchymal hemorrhage and additional hemorrhage in the right parietal region is evident.

Due to elevated intracranial pressure caused by subdural and subarachnoid hemorrhage, an osteoclastic craniotomy was performed to evacuate the hematoma and relieve pressure (Fig. 2a and b). A large volume of blood was released during trepanation, necessitating intraoperative blood transfusion. Injury to the superior sagittal sinus during the procedure resulted in significant bleeding (Fig. 3), which was managed pharmacologically with the administration of clotting agents. The excised bone flap was sent for histological analysis, which was negative for bacterial infection.

### Postoperative course in ICU

The patient remained in the intensive care unit (ICU) for ~4 weeks. A tracheotomy was performed to facilitate prolonged mechanical ventilation. Follow-up cranial CT imaging revealed a massive intracerebral hemorrhage in the right hemisphere, accompanied by a high-grade midline shift, significantly worsening the prognosis. Consequently, intensive care measures were escalated, including the implementation of a traumatic brain injury protocol. No further surgical intervention was required. The patient was eventually transferred to a general ward in a vegetative state.

### Neurological improvement and bone reimplantation

Despite the initially poor prognosis, the patient demonstrated gradual neurological improvement, likely attributable to the effective reduction of intracranial pressure. Subsequently, she was transferred to a neurorehabilitation center. However, reimplantation of the previously removed bone flap was required to ensure cranial protection before initiating comprehensive rehabilitation. The reimplantation procedure was completed successfully.

### Complication: scalp necrosis

**Figure 4:**
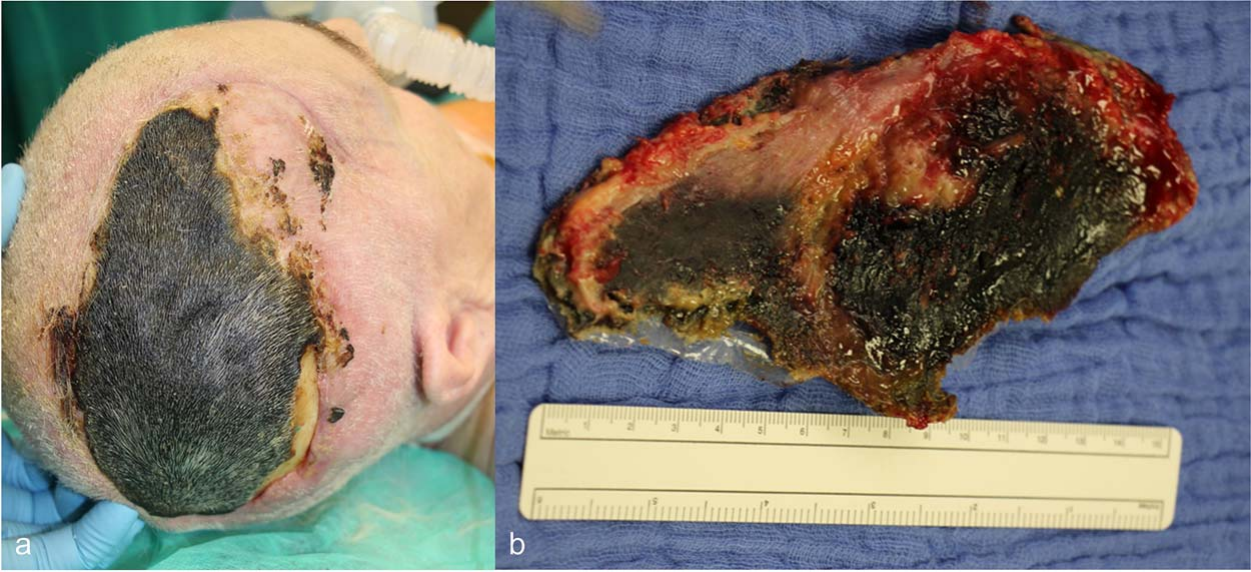
(a) Necrosis of the scalp in the area of the previously performed cranioplasty, (b) explanted necrosis area.

Shortly after transfer to the neurorehabilitation center, the patient developed scalp necrosis over the site of the reimplanted bone flap (Fig. 4a). This complication was likely attributable to impaired vascular healing, potentially related to the earlier injury of the superior sagittalis sinus. The patient was subsequently transferred back to the hospital for surgical debridement.

### Reconstructive surgery

**Figure 5:**
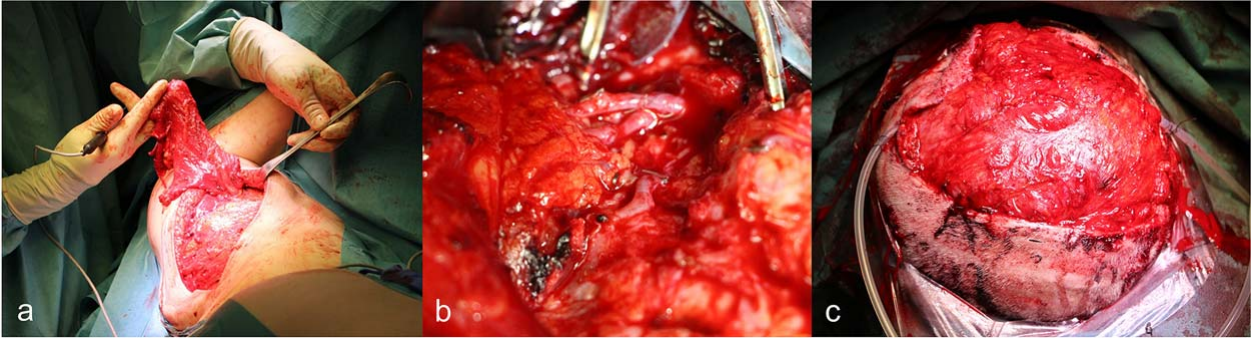
(a) Preparation of the latissimus dorsi muscle flap, (b) anastomosis of the latissimus dorsi muscle flap with the temporal artery and vein, (c) covering of the defect with anastomosed latissimus dorsi muscle flap.

**Figure 6:**
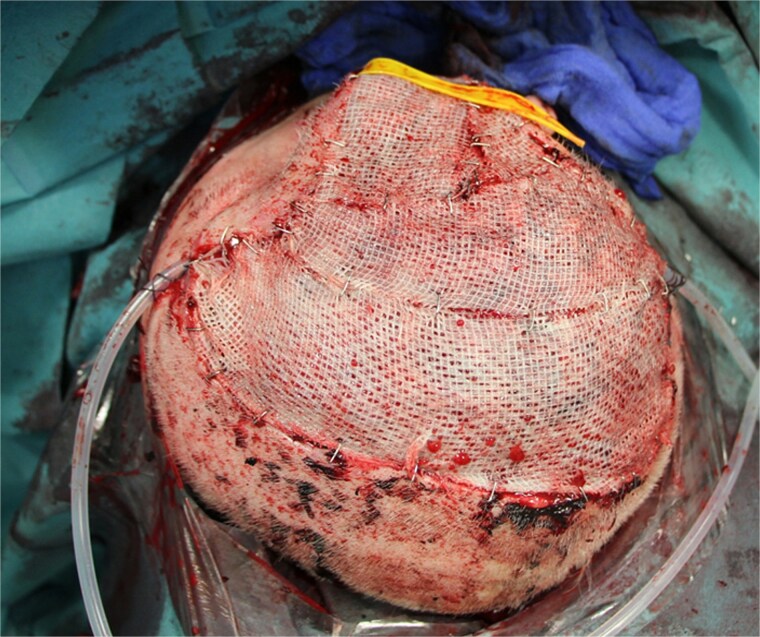
Split skin coverage including two Jackson Pratt drains.

Surgery was performed 2 weeks after the onset of necrosis. Initially, a rotational skin flap was planned. However, intraoperatively, 40% of the dura was found to be exposed following the removal of necrotic tissue and the bone flap (Fig. 4b), necessitating a change in surgical strategy. A latissimus dorsi free flap was harvested and microvascular anastomosis was successfully performed, connecting the flap’s vessels to the right temporal artery and vein (Fig. 5a–c). To complete the reconstruction, a split-thickness meshed skin graft harvested from the right thigh was used to cover the muscle flap (Fig. 6).

### Follow-up and outcome

**Figure 7:**
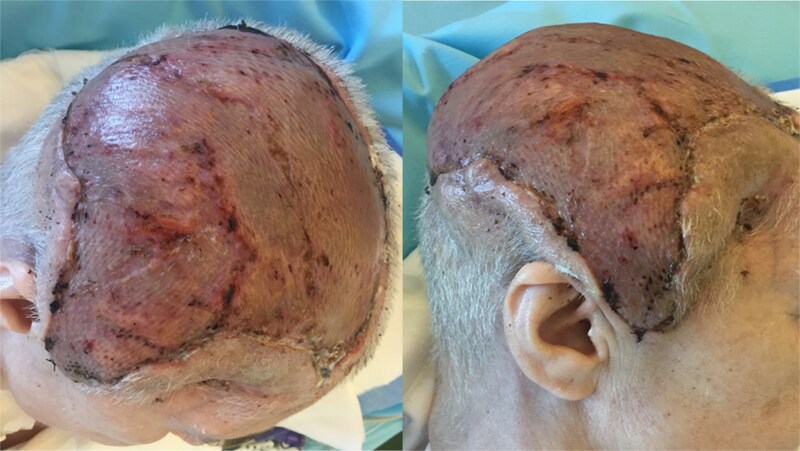
8 weeks postoperative result after latissimus dorsi muscle flap transplantation.

**Figure 8:**
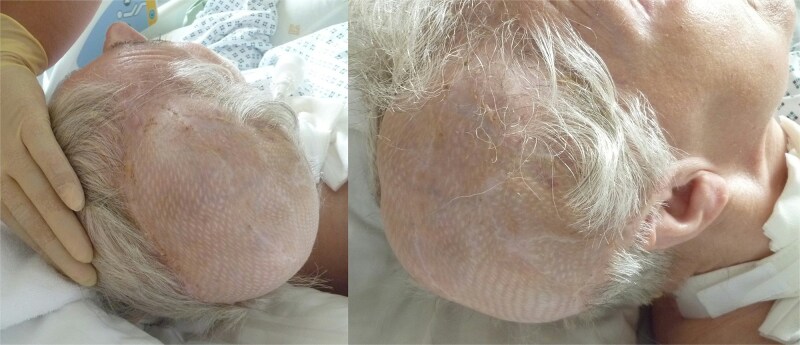
12 months postoperative result after latissimus dorsi muscle flap transplantation.

Eight weeks postoperatively, all surgical wounds had healed well, with no signs of infection or wound dehiscence. The latissimus dorsi flap provided excellent soft tissue coverage, and the reconstructed cranial region demonstrated good perfusion and structural integrity (Fig. 7). At 12 month follow-up, complete healing of the latissimus dorsi muscle transplant was confirmed (Fig. 8).

## Discussion

Although wound complications following cranioplasty are well recognized, scalp necrosis remains a rare and insufficently understood phenomenon [12–14]. In the present case, hypertension may have contributed to microvascular compromise, consistent with reports identifying it as a potential risk factor [15].

Importantly, this was not a classic wound-healing failure [16] – as the initial postoperative course was unremarkable – but rather a vascular complication likely associated with injury to the superior sagittal sinus. These findings underscore the need for careful intraoperative sinus management and vigilant postoperative surveillance.

The decision to employ a latissimus dorsi free flap rather than a simpler rotational flap was necessitated by the unexpectedly extensive dural exposure. Although more invasive, the favorable outcome in this case underscores the flap’s utility in complex cranial reconstruction [3, 4]. Furthermore, the integration of emerging technologies, such as indocyanine green angiography and patient-specific implants, holds promise for optimizing results. These adjuncts may improve flap viability and promote more precise anatomical integration [9–11].

## Conclusion

This case illustrates a rare but clinically significant complication of cranioplasty – scalp necrosis resulting from vascular compromise – and emphasizes the value of the latissimus dorsi free flap for reconstructing extensive cranial soft tissue defects. This option should be considered particularly in the setting of dural exposure or compromised scalp perfusion. Early recognition of vascular complications, combined with timely interdisciplinary management, remains essential for achieving for optimal outcomes.

## Data Availability

Data generated during and/or analyzed during the current study are available from the corresponding author on reasonable request.
